# Supplementary material for: Toward a Physiologically Relevant 3D Helicoidal-Oriented Cardiac Model: Simultaneous Application of Mechanical Stimulation and Surface Topography
Source: Bioengineering (Basel). 2023 Feb 17;10(2):266. doi: 10.3390/bioengineering10020266 (PMC9952807; doi:10.3390/bioengineering10020266)
Supplement: Supplementary file 1 [file bioengineering-10-00266-s001.zip › Supplementary 1.pdf]

Supplementary 1: DIC procedure

DIC procedure

Sample preparation

1) Fabrication of the mold, pouring PDMS unmold the chamber.
2) Apply speckle pattern with spray paint on the sample.

The pattern is a critical step of the experiment. Thus, before spraying paint on the sample,
the paint is tested on paper. Once the method that gives the best pattern is determined, it is applied to the sample. To get a well-definite pattern the dots should not overlap nor be too big, however their density has to be high enough to allow the correlation to be done. If these conditions are not fulfilled NCORR will not give any conclusive results. The computation by NCORR is only possible if the program is able identify enough points to make a correlation. Thus, as much paint as necessary should be applied, while the pattern has to be as neat as possible.

Experimental setup

A custom-built stretcher machine is used to carry on the experiment. It applies cycle of stretch to the sample. A camera is set up to focus on the sample during the cycles. A picture is taken every second, their analysis will allow the computation of the plane stress in NCORR. The sample is pinned on the grips. The machine is set to a speed of 100.

Data Collection

In order to focus the camera **Nikon D5600** (ref: https://download.nikonimglib.com/archive3/MsVU700sVgHA03pT0AR98mpYEu10/D5600UM_EU(Fr)04.pdf) on the sample, the following procedure has been fulfilled:

- Go to Menu
- Choose App list
- Choose Intervalometer
- Select a 1 second timelapse
- Set ISO to 500
- Autofocus (to get a roughly good focus)
- Manuel focus (to try to have the best focus)

This procedure requires precise manual lens aperture adjustments, an aperture of f4.0 has been used. By reducing the aperture of the camera, the angular resolution will increase but less light will pass through the lens. Increasing the aperture to its maximal value f8.0 would give better pictures; however, it would also significantly increase the process time necessary to take a picture. The picture would be blurry if the sample moves too fast.

NCORR procedure

The DIC analysis is done using the NCORR software. The different steps needed to obtain the displacement fields and
the strains fields are described underneath.

- Load the reference image and the current images
- Set the Region of Interest (ROI) for backward analysis.

It is indeed easier to adapt the ROI from the deformed image to the undeformed image than the other way around. For computational purposes, only a small region in the middle of the chamber is considered only.

- Set the DIC parameters: subset radius (15), subset spacing (3). The iterative solver default options are kept. There is no subset truncation.
- Perform DIC analysis.
- Set the format displacements: units (0.018452mm/pixel). Correlation threshold is not changed (see

Discussion). No radial lens distortion coefficient is applied.
- Set the strain parameters: strain radius (15 pixels) and lagrangian or eulerian view.

Parameter sensitivity analysis

Some key parameters need to be chosen when performing a DIC analysis. First, the subset radius gives the size of the regions where the algorithm finds the optimal deformation vector. Therefore, in theory, the subset radius has to be as small as possible in order to obtain a precise enough
mapping between the reference and deformed configuration. However, this is true without considering the noise in the images due to motion blur or simply the finite image resolution. Indeed, by taking a too small subset radius, the signal to noise ratio is decreased and the deformation vector will be more impacted by the noise. Thus, one needs to find a tradeoff between getting an accurate displacement field while keeping a good signal to noise ratio. In practical terms, one should select the smallest subset possible which does not result in noisy displacement data.
Noise can be observed for subset radius under 15. Therefore, the value 15 is selected. The
same type of analysis could have been done for the subset spacing. However, this parameter aims to reduce computational load, thus, there should not be major differences while varying it apart from the calculation time. Therefore, the default value (3) is kept.
