# Supplementary figures and images for: Toward a Physiologically Relevant 3D Helicoidal-Oriented Cardiac Model: Simultaneous Application of Mechanical Stimulation and Surface Topography
Source: Bioengineering (Basel). 2023 Feb 17;10(2):266. doi: 10.3390/bioengineering10020266 (PMC9952807; doi:10.3390/bioengineering10020266)

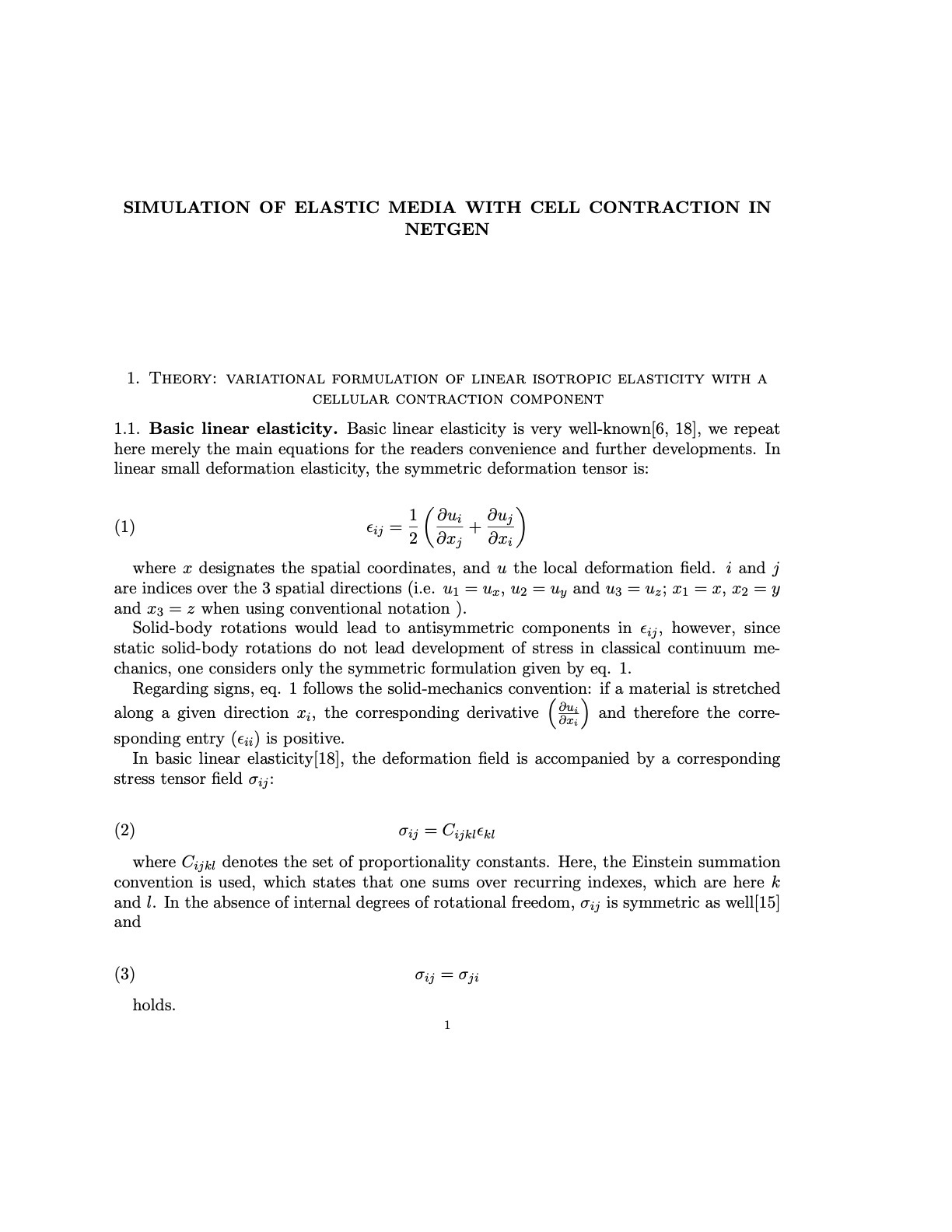

Supplement: Supplementary file 1 [file bioengineering-10-00266-s001.zip › supplementary 2_simulation_netgen copy.jpg]
